# Going upstream – an umbrella review of the macroeconomic determinants of health and health inequalities

**DOI:** 10.1186/s12889-019-7895-6

**Published:** 2019-12-17

**Authors:** Yannish Naik, Peter Baker, Sharif A. Ismail, Taavi Tillmann, Kristin Bash, Darryl Quantz, Frances Hillier-Brown, Wikum Jayatunga, Gill Kelly, Michelle Black, Anya Gopfert, Peter Roderick, Ben Barr, Clare Bambra

**Affiliations:** 1 Leeds Teaching Hospitals NHS Trust, Beckett St, Leeds, LS9 7TF UK; 2 University of Liverpool Department of Public Health and Policy, 3rd Floor, Whelan Building, Brownlow Hill, Liverpool, L69 3GB UK; 3 Global Health and Development Group, School of Public Health, Imperial College London, St Mary’s Campus, Norfolk Place, London, W2 1PG UK; 4 Department of Global Health and Development, London School of Hygiene and Tropical Medicine, 15-17 Tavistock Place, London, WC1H 9SH UK; 5 Department of Primary Care and Public Health, Imperial College London, Reynolds Building, St Dunstans Road, London, W6 8RP UK; 6 Centre for Global Non-Communicable Diseases, Institute for Global Health, University College London, 30 Guilford Street, London, WC1N 1EH UK; 7 School of Health and Related Research (ScHARR), The University of Sheffield, Regent Court, 30 Regent Street, Sheffield, S1 4DA UK; 8 NW School of Public Health, Health Education England North West, First Floor Regatta Place, Brunswick Business Park, Summers Road, Liverpool, L3 4BL UK; 9 Department of Sport and Exercise Sciences, Durham University, 42 Old Elvet, Durham, DH1 3HN UK; 10 Institute of Health Informatics, University College London, 222 Euston Road, London, NW1 2DA UK; 11 Junior Doctor and National Medical Director’s Fellow, London, UK; 12 Faculty of Medical Sciences, Newcastle University, Sir James Spence Building, Royal Victoria Infirmary, Newcastle upon Tyne, NE1 4LP UK

**Keywords:** Economy, Social determinants of health, Population health, Economic policy, Health inequalities, Macroeconomy, Public health, Regulation

## Abstract

**Background:**

The social determinants of health have been widely recognised yet there remains a lack of clarity regarding what constitute the macro-economic determinants of health and what can be done to address them. An umbrella review of systematic reviews was conducted to identify the evidence for the health and health inequalities impact of population level macroeconomic factors, strategies, policies and interventions.

**Methods:**

Nine databases were searched for systematic reviews meeting the Database of Abstracts of Reviews of Effects (DARE) criteria using a novel conceptual framework. Studies were assessed for quality using a standardised instrument and a narrative overview of the findings is presented.

**Results:**

The review found a large (*n* = 62) but low quality systematic review-level evidence base. The results indicated that action to promote employment and improve working conditions can help improve health and reduce gender-based health inequalities. Evidence suggests that market regulation of tobacco, alcohol and food is likely to be effective at improving health and reducing inequalities in health including strong taxation, or restriction of advertising and availability. Privatisation of utilities and alcohol sectors, income inequality, and economic crises are likely to increase health inequalities. Left of centre governments and welfare state generosity may have a positive health impact, but evidence on specific welfare interventions is mixed. Trade and trade policies were found to have a mixed effect. There were no systematic reviews of the health impact of monetary policy or of large economic institutions such as central banks and regulatory organisations.

**Conclusions:**

The results of this study provide a simple yet comprehensive framework to support policy-makers and practitioners in addressing the macroeconomic determinants of health. Further research is needed in low and middle income countries and further reviews are needed to summarise evidence in key gaps identified by this review.

**Trial registration:**

Protocol for umbrella review prospectively registered with PROSPERO CRD42017068357.

## Background

There has been long-standing recognition of the major role of economic factors on health and well-being [1]. There is, for example, a wide evidence base around the negative health impacts of poverty [2, 3] unemployment [4] or income inequalities [5]. At a more macro-level, there is evidence linking reductions in public sector spending with health inequalities [6] and seminal commissions have recognised the role of social protection, taxation and gross domestic product (see for example [7, 8]).

Previous umbrella reviews have, for example, considered economic interventions such as taxes and subsidies and individual level interventions such as income transfers [9], the effects of “public health policies” such as taxation [10] and of broader political factors [11], highlighting the importance of a wide range of economic factors on health outcomes. Thomson et al. suggested that tobacco taxation was not supported by evidence whereas controlling the advertising of tobacco was supported, and finding evidence to support taxes on unhealthy food and alcohol. McCartney et al. concluded that social democratic welfare states, higher public spending, fair trade policies, compulsory education, micro-finance initiatives, health and safety regulation, universal access to healthcare, and high quality, affordable housing have positive impacts on health whilst the retrenchment of the public sphere associated with neoliberalism has negative effects.

However, amidst continued concern around economic inequality [12] there is still a lack of conceptual clarity around the macroeconomic determinants of health and there is no comprehensive evidence regarding policies or interventions to address them. This is particularly pertinent in light of policy debates about Health in All Policies—a move to consider the impact on health and health inequalities in all aspects of government policy [13].

This review aims to provide a conceptual model to understand the links between the macroeconomy and health, and a systematic umbrella review of the systematic review evidence base in this field, examining the links between macroeconomic determinants, and health and health inequality outcomes. The review protocol was published [14] and registered with PROSPERO, the International Prospective Register of Systematic Reviews (CRD42017068357). This review thus provides evidence to policymakers, researchers and health advocates that can be used to develop evidence-based economic policy interventions and clarify priorities for further research.

## Methods

This paper provides a summary of the methods and clarifications to the protocol (Naik 2017).

### Research question

What are the effects of macroeconomic factors, strategies, policies and interventions on population health and health inequalities?

### Conceptual model

The economy has been defined as a ‘social domain that emphasizes the practices, discourses, and material expressions associated with the production, use and management of resources’ [15]. The economy is thus perceived as a complex interacting system which influences health through a number of mediators (access to healthcare, housing, etc.) and in interaction with other determinants such as social and environmental factors.

**Fig. 1:**
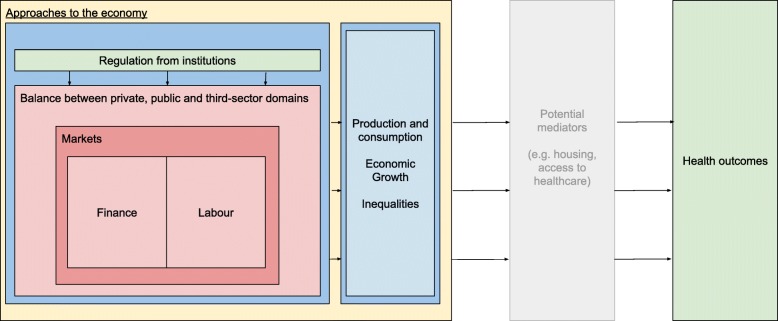
Conceptual model of links between the economy and health. (Reproduced from protocol)

The *Journal of Economic Literature* (JEL) provides a classification [16] of the key concepts that relate to research in economics. Based on the JEL terms, it is proposed that the economic factors that influence health can broadly be perceived in seven major categories—market regulation; institutions; supply of money; finance and loans; the balance between the public, private and third sector; labour; production and consumption and approaches to the economy. Table 1 presents these seven categories, with related subtopics for each category at the local, national and international level. Whilst this list is not exhaustive, it provides an initial framework to guide the search strategy. An a priori framework (Fig. 1) is also proposed to show the broad relationships between economic factors and health outcomes.

**Table 1:** Matrix of economic factors at local, national and international level (Reproduced from protocol)

	Local level	National	International
Category 1: Market regulation		Competition including legislation, consideration of externalities in pricing, fiscal measures e.g. tax, market structure, trade regulation	Trade policy
Category 2: Institutions		Central bank, banks, micro-finance, mortgages, startups. Legislation and regulation of organisations	International organisations e.g. IMF, World Bank, multinational firms, World Trade Organisation
Category 3: Supply of money, finance and loans	Local currencies, debt	Interest rates, inflation, deflation, wages, supply of money or credit, macroeconomic policy, fiscal policy, financial crises, monetary policy, structural adjustment policies, natural resources	International lending, foreign aid, Financial transactions tax, capital controls
Category 4: Balance between public, private and third sector	Land tenure, informal economies, shadow economies, social enterprises and cooperatives	Structure and scope of government, privatisation and nationalisation, taxation, tax avoidance, government expenditure and welfare provision, property rights	
Category 5: Labour	Firm governance, structure, ownership, behavior,	Trade unions, employment, unemployment, minimum wage, labour force size and structure	
Category 6: Production and consumption	Income, wealth, distribution	Industrialisation, economic growth and aggregate productivity	
Category 7: Approaches to economy	Regional economics	E.g. Capitalist, socialist, transitional, Keynesian, Marxian, Neoclassical, ecological economics

### Design

An umbrella review (a systematic review of systematic reviews [17]) was carried out to synthesise evidence across multiple reviews and thus provide a seminal overview of evidence in the field. Umbrella reviews are an established method of locating, appraising and synthesising systematic reviews of interventions [18]. They use systematic review methodology to locate and evaluate published systematic reviews of interventions. Umbrella reviews are therefore able to present the overarching findings of such systematic reviews [19]. In this way, they represent an effective way of rapidly reviewing a broad evidence base [17]. This transparent approach to retrieving robust evidence is increasingly common in the field of public health [10, 20]. A PRISMA statement is included in Additional file 1.

### Search strategy

Lead researchers developed a pilot search strategy with the help of an information specialist and experts. The search included a combination of economic terms developed from the JEL classification system [16], health outcomes, from a previous umbrella review, [20], and a modified version of the Scottish Intercollegiate Guidelines Network systematic review filters was used for health equity search terms [21]. The search was tested on Medline to ensure selected “tracer papers” were found as expected, in line with previous umbrella reviews [18]. See Additional file 2 for example search strategy.

A search was carried out using the following databases: Medline (Ovid), Embase (Ovid), Econlit (EBSCO), PsycInfo (Ovid), Applied Social Sciences Index and Abstracts (ASSIA; ProQuest) and Sociological Abstracts (ProQuest) for relevant papers from 3/11/18 to 11/11/18, developing tailored searches for each database. Post-protocol, a decision was taken to also search the Cochrane Database and Database of Abstracts and Reviews (DARE) for completeness. A key review from each category was hand-searched for further citations (*n* = 6). Two important umbrella reviews were published following the search – [10, 11] both were citation searched for other reviews as they had significant overlap in scope with this umbrella review.

### Inclusion criteria

A robust set of inclusion criteria was developed (Table 2). In summary, these focused on the retrieval of peer reviewed systematic reviews exploring the impact of macro-, population-level economic factors on health and inequalities outcomes, with specific mediators such as healthcare policy or other social determinants of health being excluded. Only reviews in English were included.

**Table 2:** Criteria for including systematic review articles, in the present umbrella review (modified from protocol)

Study design	Systematic reviews meeting Database of Abstracts of Reviews of Effects (DARE) criteria: (i) a defined review question (which includes at least two out of population, intervention, comparison, outcomes or study designs), and with a search strategy of a named database, and (ii) a search strategy including both a named database (at least) and one of the following: reference checking, hand searching, citation searching or contact with authors [22].
	No restrictions to specific types of primary study designs. Reviews of both interventions and associations were included.
Timeframe	No restriction
Population	Adults and children
Intervention/exposure	The reviews focused on macro-, population-level economic factors falling into the 7 categories outlined above.
	Post protocol clarification – Studies at the international/ national / regional or municipal level. Studies investigating individual-or neighbourhood level factors were excluded.
Comparator	Studies with and without controls
Outcome	Health and health inequality outcomes. Primary outcomes including but not limited to morbidity, mortality, prevalence and incidence of conditions and life expectancy. Secondary outcomes including health inequalities by gender, ethnicity or socio-economic status (for example by income, education, employment, receipt of benefits at an individual or area level). Cost-effectiveness data was also extracted if available.
Setting	Any setting—low, middle, high-income countries.
Year considered	All years since the start of database until the search date (searches run from 3/11/2017 to 11/11/2017)
Language	English language
Publication status	Only peer-reviewed published studies

### Study selection and data extraction

Titles and abstracts of papers were screened by a member of the research team to exclude irrelevant papers, with a random 10% sample being independently screened by a second author [23]. A screening tool based on the inclusion criteria was used in this stage (see Additional file 3). There was high agreement in this first phase of screening (96.5%), and a precautionary approach was taken which involved including studies unless they clearly warranted exclusion. Disagreements were resolved through arbitration by a lead author and tracer papers were automatically listed for a full text review. A kappa score was not calculated as this statistic is not appropriate in cases where a positive result is a rare event (as was the case for the number of inclusions relative to the number of papers found).

A full text screen was then conducted independently by two different members of the research team, with 94% agreement and the remainder requiring arbitration by a lead author. Due to a variation in percentage agreement across screening pairs (94, 96 and 88%), a further review of all papers selected for inclusion was carried out by a lead author and any further exclusions agreed between two lead authors to ensure consistency in the application of the exclusion criteria. Additional file 6 includes a list of all papers included at full text screen.

Studies were excluded if they focused on individual-level exposures or interventions, focused solely on mediators (including the organisation or access of healthcare) ordealt solely with health-related behaviours or risk factors as outcomes (e.g. smoking or obesity). Studies were also excluded if they conducted a systematic review of a health topic, and then added macroeconomic data to conduct further analysis or modelling based on linking this data with the results of the systematic review; we considered these to be effectively primary research. A pragmatic decision was taken to exclude small, neighbourhood area-level factors as exposures as they can be classed as meso- rather than macro-level economic factors.

Key data was extracted from included papers using standard extraction forms adapted from previous reviews for this purpose [23] (see Additional file 4) Where not all of the findings of a review were relevant to our scope but the review included a small number of relevant findings, we have included the relevant findings. Data extraction was conducted by single authors and checked by a lead author at writeup stage.

### Quality appraisal and data synthesis

The reviews selected were quality appraised using the Assessment of Multiple Systematic Reviews 2 (AMSTAR 2) approach [24] with all relevant data from the AMSTAR checklist being extracted on a separate proforma as part of the data extraction. This critical appraisal tool, developed to facilitate the assessment of systematic reviews, guides the user to explore study selection and extraction, search details, methods of synthesis, assessment of publication bias and conflict of interest. It is an update on the original AMSTAR which has become standard as part of umbrella review methodology [10]. AMSTAR 2 is designed to be modified for each study. In this study, criterion 7 of AMSTAR 2 (“Did the review authors provide a list of excluded studies and justify the exclusions?”) was only counted as a weakness rather than a critical weakness as it was felt that this feature would not necessarily be present in the retrieved reviews spanning a broad range of disciplines and journals. Partial weaknesses on critical criteria were also classed as critical weaknesses, as there was no clear guidance on how to handle such cases. In line with AMSTAR, quality appraisal was focused at the review level and not at the individual study level. Where a quality appraisal of the underlying primary evidence had been carried out by the review authors, this information was also extracted.

Where a meta-analysis had been carried out, the combined effect size was reported. Where the review did not provide a summary measure of effect, key findings were used to inform a narrative overview. No meta-analysis was carried out as part of this review, given the broad topic being studied and the heterogeneous nature of the included material. A narrative overview of the findings was presented to ensure a description of the underlying evidence, based on discussion amongst authors. All authors reviewed the final content to ensure that the paper reflected the underlying evidence base.

## Results

**Fig. 2:**
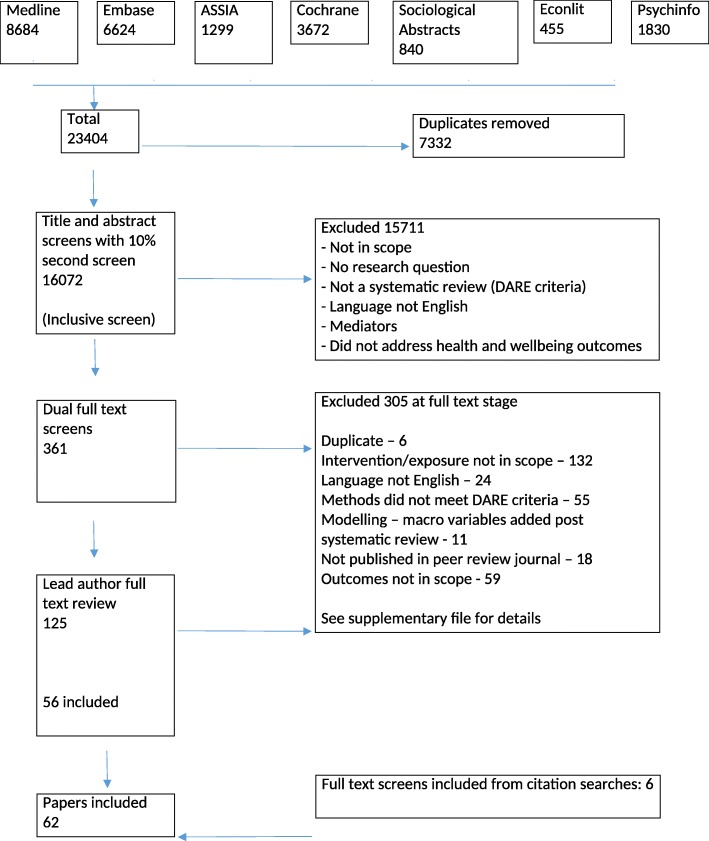
Flowchart

In total, 62 reviews were identified for inclusion in the umbrella review. Figure 2 shows the flowchart for the screening and inclusion. These were classified according to their main area of focus, though some papers were relevant to multiple categories. Table 3 shows the number of papers included in each category or crossing category boundaries and the number of reviews scoring each level of quality from the AMSTAR 2 checklist.

**Table 3:** Number of papers included in each category

	Number of reviews in category	AMSTAR 2 High	AMSTAR 2 Moderate	AMSTAR 2 Low	AMSTAR 2 Critically low
1: Market Regulation	13	2	2	1	8
2: Institutions	0	0	0	0	0
3: Supply of Money, Finance and Loans	11	0	0	5	6
4: Balance between public, private and third sectors	7	0	0	4	3
5: Labour	6	1	2	0	3
6: Production, Consumption and Distribution	12	0	0	1	11
7: Approaches to Economy	5	0	0	0	5
Papers spanning category boundaries	8	1	1	0	6
Total	62	4	5	11	42

### Description of included reviews

The reviews comprised a range of methodologies including umbrella reviews, meta-analyses and narrative reviews and were mostly of critically low and low quality by AMSTAR 2. Often the macroeconomic exposure of interest was only a part of the review and therefore a subset of relevant findings was extracted. The majority of reviews focused on high-income countries or middle- and high-income countries, and much of the underlying evidence base was made up of observational studies including cohort studies and cross-sectional studies. There was some limited use of intervention and modelling studies. Reviews deployed a range of approaches to assessing quality in their underlying studies. Common weaknesses included a lack of consideration of quality or bias, an unstructured discussion of these issues, and a range of different structured tools to appraise retrieved studies. Overall, the AMSTAR quality of the evidence base was low with only *n* = 9 reviews of a high or moderate quality rating.

### Overview of results

**Fig. 3:**
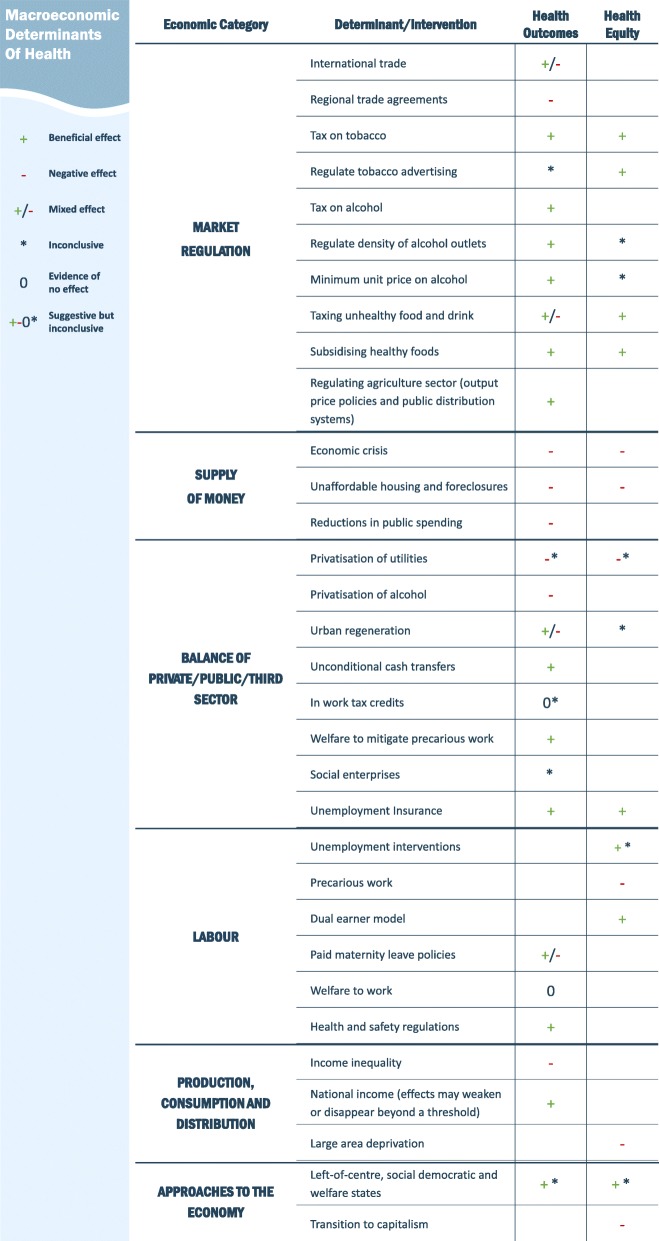
Overarching summary of findings

The findings underscore a complex and uneven evidence base around the macroeconomic determinants of health, characterised by several specific topics with a substantial evidence base in systematic review format such as the impacts of economic crises and the market regulation of health related goods, and large areas of the field without systematic review-level evidence such as the role of institutions in regulating the economy. Figure 3 below shows an overarching summary of the findings, describing the health and inequalities impact of different determinants and interventions. A summary of each paper including summary findings and description of underlying study quality is provided in Additional file 5 whereas the below description covers only the main findings in narrative form.

### Category 1: market regulation

#### Tobacco

Three reviews explored the regulation of the tobacco market. A review of reviews of critically low quality found mixed evidence around the health equity impact of tobacco pricing and a lack of evidence around advertising restrictions [25]. A more recent high quality review suggested that restricting advertising was likely to have a neutral equity impact whilst taxation was likely to be pro-equity [26]. Another moderate quality review [27] found mostly positive but mixed evidence for taxation on child health outcomes such as infant mortality and preterm births, with reductions in preterm births among mothers with low education and black mothers.

#### Food, drink and agriculture

Five reviews of critically low quality explored the role of food and drink taxes or subsidies, mostly within middle to high income countries. Several reviews found limited evidence around the end health outcomes related to food and drink taxes or subsidies [28–30]. A 10–15% tax was highlighted as a minimum to achieve positive outcomes [29]. On the other hand, Thow et al. found the combination of taxing unhealthy food along with subisiding fruit and vegetables was likely to reduce cardiovascular disease incidence [31]. Eyles et al. highlighted that some high quality studies showed adverse non-communicable disease outcomes from taxation due to substitution and suggested that taxes and subsidies used together could have a positive impact [32]. These interventions were also found to be pro-equity [30, 32].

In a broader review of reviews of moderate quality, Galvao et al. identified several agriculture interventions, such as output prices policies and public distribution system policies, to be associated with positive health outcomes [33].

#### Alcohol

Five reviews of differing quality focused on alcohol, mostly in high income countries. Higher alcohol taxes and pricing were found to be associated with many positive outcomes including lower motor vehicle mortality rates, premature mortality, suicide and cirrhosis mortality in a critically low quality review [34]. A high quality review found alcohol taxes to be associated with reduced alcohol related harm, and recommended a tax of at least 10% [35]. Through a moderate quality meta-analysis Wagenaar et al. found that doubling alcohol taxation may be associated with a decrease in alcohol-related mortality by 35% and a range of other smaller improvements in health outcomes [36]. Boniface et al. found that Minimum Unit Pricing was likely to reduce harm from alcohol in a low quality review [37].

In an umbrella review of critically low quality Martineau et al. concluded that there is clear and consistent evidence that taxation reduces alcohol-related harm, and that regulating the alcohol availability within areas may reduce harm dependent on surrounding areas and context [38].

Privatisation is also considered within the evidence base on alcohol markets, which is dealt with under a separate category in this current review [38, 39].

#### Trade

Two reviews of critically low quality dealt with trade. The first found that higher levels of international trade, Foreign Direct Investment or higher globalisation scores were likely to be associated with better population health, but also highlighted risks due to communicable diseases, pollution and insecure contracts [40]. Barlow et al. focused on regional trade agreements, finding evidence that these were associated with higher cardiovascular disease incidence though with mixed evidence around infant mortality, life expectancy and tuberculosis incidence [41]. This evidence was of moderate to high quality and was mostly related to low/middle income countries or to a global context. The reviews support the idea that there is a multi-tiered pathway between trade agreements and health outcomes, mediated by health services and policy, as well as production and consumption patterns.

### Category 2: institutions

No reviews were found addressing the health and health inequalities impacts of economic regulation from key institutions.

### Category 3: supply of money, finance

#### Economic crisis

Ten reviews included in this category were of low or critically low quality and focused on the impacts of economic crisis. There was significant heterogeneity in definitions of crisis used which included the 2008 crisis and broader factors such as population level employment or Gross Domestic Product (GDP) changes and policy responses, with most reviews focusing on high income countries.

Evidence was mixed around mortality [42, 43]. An increased prevalence of diabetes, asthma and cardiovascular disease [44] as well as mental health and suicide [43] has been documented. There was evidence to suggest a higher exposure to infectious diseases and associated rise in mortality, though this was mixed and at risk populations such as prisoners and migrants were most likely to experience adverse outcomes [45]. There were conflicting results on child mortality across the world [46]. One study found a range of worsening health outcomes in Greece but causality was limited [47]. There was conflicting evidence of differential impacts by gender and age and income appears to be a key factor in mediating the resulting impact [43, 44, 46, 48]. Herbig et al. also considered the evidence around unemployment as part of economic crisis, however the findings of this review are primarily reported on in the labour category [49].

There was conflicting evidence of the impacts of economic crises on alcohol-related harm with a possible widening of health inequalities [50]. Kentikelenis et al. cite evidence that economic crises may lead to unemployment or substance use amongst migrants to suggest a greater vulnerability to infectious disease amongst this population group, but there was inconclusive evidence around end health outcomes [51]. Two reviews highlighted primary research suggesting that economic crises may be partly acting through policy choices to reduce public spending on services [45, 51].

#### Housing foreclosure crises and unaffordable housing

Two reviews were included focusing on these topics which were deemed primarily economic in origin [52, 53]. They were of low and critically low quality and mostly considered evidence from the United States. These studies found that overall these factors were associated with worse mental health. The findings were conflicting around suicide and suggested that there may also be negative physical health impacts though evidence on the latter was limited. It was highlighted that these events can affect areas already experiencing deprivation thus widening health inequalities.

### Category 4: balance between public, private and third sectors

#### Privatisation

Three reviews covered the effects of privatisation on health. One high quality review [39] found limited evidence suggesting significant association between privatisation and an increase in alcohol related harm or between remonopolisation and a reduction in alcohol related harm. These findings are consistent with those of another review retrieved in a related umbrella review of critically low quality [38]. Egan and colleagues found modest evidence of a decline in psycho-social wellbeing among employees following privatisation in their sector, and inconsistent evidence against other health outcomes in a low quality review [54].

#### Urban regeneration

One critically low quality review reported limited evidence of a paradoxical reduction in self-reported wellbeing after the implementation of an urban regeneration programme, and small reductions in overall mortality rate [55].

#### Subsidies and unconditional cash transfers

One low quality review of heterogeneous studies found a small increase in mean birthweight among children born to mothers who were food subsidy programme participants [56]. Pega and colleagues, in a review of low quality and a small number of studies, found clinically significant reductions in the risk of child death among those in receipt of unconditional cash transfers in humanitarian settings [57].

#### Welfare

One critically low quality review found no systematic evidence of positive effects of in-work tax credits on health outcomes [58]. One critically low quality review linked a large supportive welfare state with a lower likelihood of experiencing negative health outcomes relating to precarious work and job insecurity [59]. Generous unemployment insurance was found to be associated with better subjective wellbeing in the general population and to mitigate the negative impacts of unemployment in a critically low quality review [60]. Additionally, another review of critically low quality found that welfare had only a weak association with health inequalities [61].

#### Social enterprises

Roy and colleagues looked at the relationship between social enterprise and health outcomes in a low quality review and found only impacts on intermediate outcomes and no evidence of an impact on health inequalities, though social enterprises may reduce marginalisation of vulnerable groups [62].

### Category 5: labour

#### Promoting employment

Three reviews covered the effect of employment on health outcomes, all of critically low quality. Three papers focussed on overall area-level employment rate and health: one meta-analysis of a small number of ecological studies showed an increase in suicide from increases in population-level unemployment, though noting insufficient evidence to draw clear conclusions [63]; another review [49] identified decreases in employment rate linked with worse health outcomes including suicide, cardiovascular disease, infectious diseases and homicide though decreases in accidents; whilst a third review of evidence, mostly from time series studies, found an association between unemployment rate and mortality due to cardiovascular disease, though mixed evidence around the link to road traffic accidents [64].

One high quality review found that welfare to work – financial incentives, training and childcare subsidies – initiated at government level had mixed effects of a magnitude that was unlikely to have health effects [65]. In a critically low quality review, Herbig et al. found evidence that an active labour market programme – the government intervening to help the unemployed find work – could mitigate the impact of unemployment on suicide [49].

#### Gender equity in employment

Four papers of mixed quality reviewed gender issues relating to labour and health. Paid maternity leave was ambiguously associated with health impacts in one moderate quality review [66], where studies reviewed at policy level found no association or suggested a negative association between maternal health and paid maternity leave. Borrell and colleagues, in a paper of critically low quality, reported evidence of an association between the “dual-earner policy model” which encourages equity in the labour market including when work is combined with parenting, and is associated with the Nordic welfare model – and positive maternal outcomes although this was in the context of other gender inequalities in health [67]. As a secondary finding, one moderate quality review found that precarious work created gendered patterns of health inequalities [33]. In a review of critically low quality, Kim et al. found a complex evidence base suggesting that vulnerability to precarious work or job insecurity was dependent on gender and that this relationship varied depending on the nature of the welfare regime [59].

#### Working conditions

Suri and Das report a decline in occupational injury incidence in India to 0.9 per 1000 (2011) from 66 per 1000 (1980); but 10% of these were fatal in 2011 compared to 0.2% in 1980; women, children and informal workers were likely to face greater impacts in this critically low quality review [68]. A moderate quality review also supports population level interventions such as occupational health and safety regulations and preventing exposure to toxic chemicals, in reducing health inequalities [33]. Bambra and colleagues found evidence that the safety regulations in the construction industry may be associated with lower fall injury rates in a critically low review [19], citing a moderate quality review by Rivara et al. [69].

### Category 6: production, consumption and distribution

#### National income

Two reviews of critically low quality were included that dealt primarily with national income. Iemmi and colleagues found no relationship between national income and suicide rates [70]. On the other hand, O’Hare and colleagues found that a 10% increase in GDP per capita would result in a 10% decrease in infant mortality though they also found that this effect was stronger for middle income countries compared with high and low-income countries and therefore suggested the traditional Preston curve^1^ as a sigmoid curve [72].

In another review of critically low quality considering the role of the welfare state in moderating health outcomes as a result of economic inequality, Kim found conflicting evidence regarding an association with GDP beyond a threshold [73].

#### Income inequality

Eight reviews of critically low quality and one review of low quality were included that dealt with income inequality. These drew on a wide range of underlying studies from a range of different contexts. In a review in 2003, Macinko et al. found it difficult to draw definitive conclusions about the relationship between income inequality and health [74]. Overall, 33 analyses showed a statistically significant association between higher income inequality and poorer health outcomes, while 12 studies showed no such relationship and some of the more sophisticated studies showed negative findings. There was mixed evidence for each type of health outcome. At a similar time, Spencer et al. found that various measures of income inequality were associated with both increased infant mortality rates and low birth weight [75].

More recently Kondo et al. found evidence of a modest effect of income inequality, calculating a relative risk for mortality per unit increase in Gini coefficient of 1.08 (95% CI 1.06 to 1.1) and an odds ratio for poor self-rated health of 1.04 (95% CI 1.02 to 1.06) [76]. The effect was found to be more strongly associated with a higher Gini which supports the idea of a threshold beyond which effects appear. On the other hand, another review found that the association with subjective wellbeing was not statistically significant *r* = − 0.01 (95% CI − 0.08 to 0.06) [77]. Another recent review by Kim et al. suggests that income inequality and health were not commonly found to be related except in terms of infant and child mortality [73]. Another review found that relative deprivation measured using the Yitzhaki index is associated with worse mental health, all-cause mortality, self-rated health, and other physical health outcomes such as birth outcomes, and functional disability [78].

Singh et al. found an association between income inequality and oral health outcomes, however the specific findings of this review are not well described as the study focused more on theoretical mechanisms of effect [79]. Costa et al. found evidence in agreement that Gini impacted on dental health outcomes [80]. Another low quality review found one study supporting an association between county level income inequality and depressive symptoms [81].

#### Large area level socioeconomic status or deprivation

A low quality meta-analysis explicitly exploring the effect of large area-level socioeconomic status found that areas with lower socioeconomic status had a relative risk of mortality 1.10 (95% CI: 1.06–1.15) times that of those with high socioeconomic status [82]. The association between large-area deprivation and health outcomes is complex; in a review of critically low quality Baade et al. highlighted a study that showed that prostate cancer mortality was associated with small area deprivation whilst the reverse was true at the county level [83].

### Category 7: approaches to the economy

Six reviews of critically low quality dealt with the underlying approach to the economy, considering evidence from a wide range of countries and significant heterogeneity in classifications of exposures. Brennenstuhl et al. discussed multiple methods of welfare regime typologies finding that health and health inequalities outcomes were inconsistently associated with the welfare regime [84]. A more recent review suggested Scandinavian welfare systems had better infant mortality but not other outcomes [73]. The findings from Borrell et al. related to the “dual earner policy” of the Nordic welfare states are reported in the labour category above [67].

Berqvist et al. explored several ways of exploring the relationship between the welfare state and health outcomes including classifying countries by regime, institutional policies and expenditure [85]. They found that the regime approach provided no consistent findings, whereas using the institutional and expenditure approaches provided more conclusive findings. In particular their findings suggest that generous welfare policies benefit all residents, and that greater health and social care spending is associated with better population health and reduced health inequalities.

In the most recent review, dealing with 176 studies, Barnish et al. found that the majority of evidence suggested that welfare states were likely to be associated with better child mortality, general health, infant mortality, life expectancy or adult mortality and reduced health inequalities. They also found that left of centre political traditions were likely to be associated with better life expectancy, infant or adult mortality and possibly a range of other outcomes though there was limited evidence around these [86].

One review found that rapid transitions from planned to free market political economies, or transitions to more neoliberal economies were associated with a worsening of health inequalities and that there was a weak association between welfare states and health inequalities [61].

### Topics with no systematic reviews

A number of topics which formed a core part of our original conceptual model were not covered by any systematic reviews. For simplicity, these are summarised below in the section on the future of research in this field.

## Discussion

In assessing a wide range of macroeconomic factors which influence health, this review has found a large (*n* = 62) but low quality systematic review evidence base on the effects of macroeconomic factors on health and health inequalities. Whilst the results can therefore only be tentative, some clear findings do emerge from our overview of the systematic reviews.

### Summary of results

First, this review found evidence that regulating the market for health-related goods through strong taxation and subsidisation is likely to be effective in improving health and reducing health inequalities. There is also evidence to support other interventions such as reducing availability or changing production patterns. International trade policies have a complex association with health outcomes involving both potential benefits and risks.

Work remains a core determinant of health, yet the evidence linking employment at the population level with health outcomes remains limited. There is evidence to support the importance of policies that promote employment and legislation to improve working conditions. Issues of gender equity are important and interventions such as dual earner policies may help – although the evidence base is small and inconclusive.

There is evidence to support the role of welfare provision in mitigating the impacts of precarious work and of cash transfers and subsidies to improve health. There is also evidence highlighting the potential for privatisation to worsen working conditions and alcohol related harm. There is limited or inconclusive evidence addressing other approaches including the role of social enterprises, in-work tax credits and welfare to work programmes.

On balance, it appears that the effects of economic crises are associated with detriments to health and health inequalities in the longer term, though the evidence base is complex and conflicting, partly due to the multiple processes that can be involved in an economic crisis including declines in national income and employment. The outcomes also depend on the context and policy response to the crises. There is also evidence that housing foreclosures and unaffordable housing have negative health impacts.

It is also hard to draw firm conclusions about approaches to the economy and the welfare state given the diversity of exposure classifications used, though there is evidence that generous welfare states and left of centre political traditions may be associated with better health outcomes and lower health inequalities. Rapid transitions to capitalism appear to have a negative impact on health inequalities though the long-term implications are unclear. The reverse transition to generous welfare states may reduce health inequalities.

The last few years have seen a growing and increasingly nuanced evidence base on macro-level economic inequality and national income which are both associated with health outcomes. Further work is needed to explore this and other dimensions of production, consumption and distribution, with a key focus on interventions to address economic inequality and optimise the health impacts of macro-level economic development. Specific topics where no systematic review level evidence exists constitute one of the strongest findings of this review; these are described below in relation to the future of macroeconomic determinants research.

### An updated conceptual model and connections between factors

The evidence highlights the importance of seven key macroeconomic determinants of health on population health or health inequalities: i) type of economy and national income, ii) economic crisis with decline in GDP, rise in unemployment and policy responses, iii) the provision of welfare, iv) the labour market, access to work and working conditions v) the balance of privatised, nationalised and social economies, vi) the market regulation of health-related goods and international trade and vii) population-level income and inequality. These can be considered as interconnected factors, each linking directly to health outcomes and health inequalities.

**Fig. 4:**
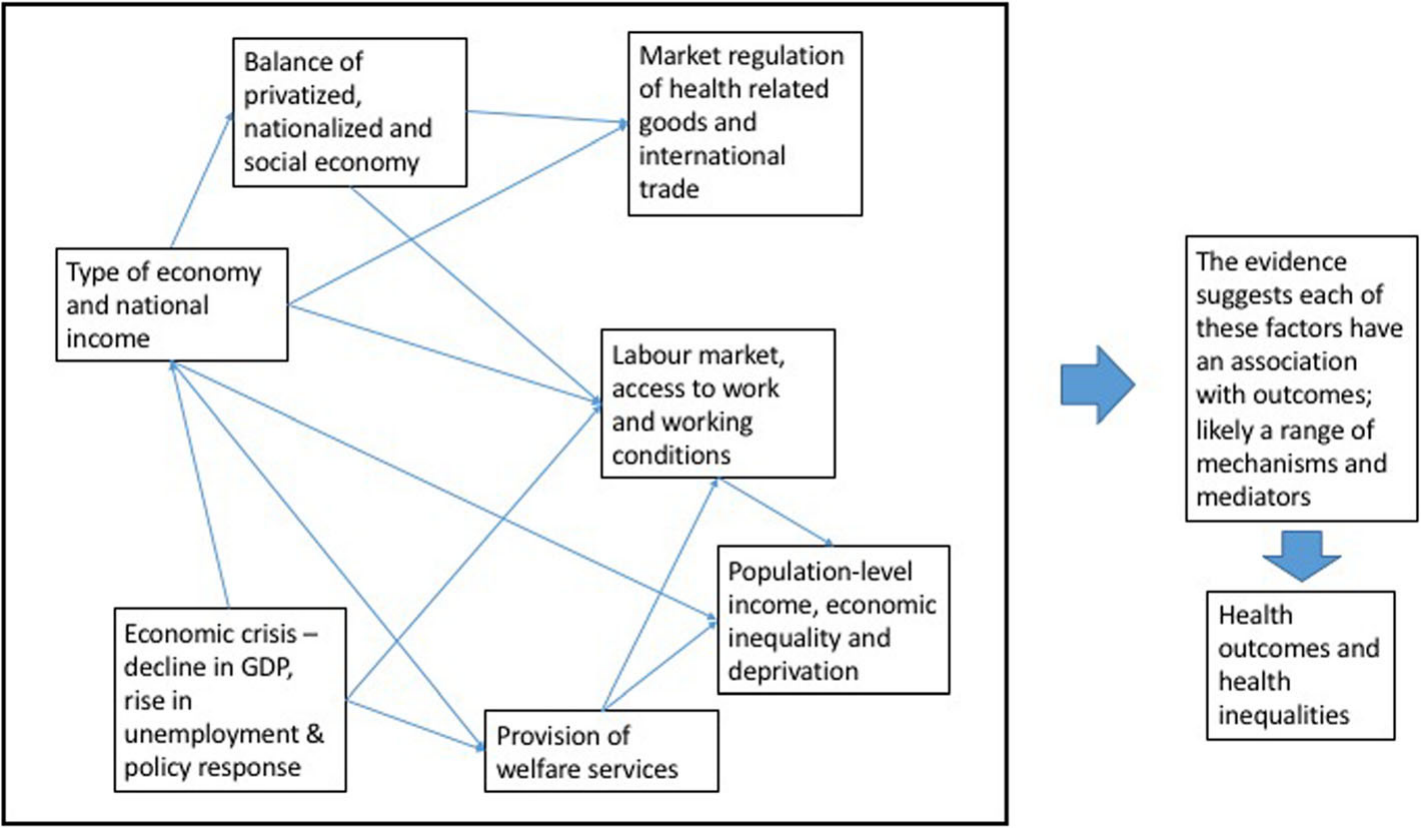
Simplified conceptual model based on review findings

A simplified model providing an overview of this level of evidence is shown in Fig. 3 below. Given the conceptualisation of the economy as a complex system, it is inevitable that the identified variables connect with each other; some of these interconnections are described illustratively below and in Fig. 4 (note that this review has not explored interconnections between factors but some of these are described in the retrieved papers). Whilst this review has not systematically identified these connections, some of these are made explicit in the retrieved reviews. For example, the underlying approach to the economy is intrinsically linked with policies around welfare. Reviews looked at the role of welfare in mitigating the negative health impacts of insecure work, or of promoting access to work [59, 65]. The process of economic or financial crisis can be considered as an acute shock involving changes to employment and national income.

Other connections may be inferred. For example, economies that are more focused on conducting international trade may be less willing to implement strong market regulation in health-related sectors. The amount of trade liberalisation a country experiences and the extent of market regulation of health-related goods are likely to be closely linked to the approach to the economy, the type of economy and regulation by international institutions. Patterns of job availability represent structural and variable factors driven by economic policies and changes in demand and supply in markets.

### Findings in context

This review indicates that there is good evidence for regulating tobacco, alcohol and food markets and for policies that promote employment and improve working conditions. Our review complements the findings of two other recent umbrella reviews. The first, on public health policies and their impact on health inequalities, included a focus on policies to reduce consumption and harm from alcohol, food and tobacco [10]. The findings are largely in agreement with those of this review – that the regulation of these goods is likely to reduce health inequalities; though this review has been able to identify more evidence specifically focused on health outcomes.

The second related umbrella review, by McCartney and colleagues, reviewed political economy factors including differences or changes in policy, law, rules, economic conditions, institutions, social structures, politics, power or conflict [11]. There was some overlap in terms of the papers included in the McCartney et al. review and those in this review. However, due to differences in remit (political economy v economy), methods and inclusion criteria as well as different search dates, there are some key differences in the final inclusion papers and the conclusions drawn. For example, McCartney et al. included papers that explored factors at the individual level around the role of microfinance and trade, and papers exploring the role of health policy. Whereas this review has not included these, it has retrieved a larger number of reviews focused explicitly on the macroeconomic determinants of health, due to our tailored search across multiple databases. Given the conceptual framework that underpins this review, it has also been possible to clearly identify research gaps in relation to this framework. Both reviews are largely in agreement around the importance of these economic factors and the need for further research though there are differences in how the findings are synthesised – for example, this review adopts a more critical view of the evidence around welfare state exposures, finding the evidence base complex and potentially inconclusive given the variety of exposure classifications used.

This review has found an absence of review level evidence to inform other major policies that influence the economy e.g. land reform, monetary policy, the role of economic institutions, tax and benefit systems. There is however growing interest on these policies particularly in debates about inclusive growth and inclusive economic development [87, 88].

For example, there has been rising interest in the social and solidarity economy – a collection of organisations and businesses that promote community solidarity and social benefits as well as producing goods and services, and which has significant potential health benefits [89]. This is closely linked to emerging work around the role of large anchor institutions within the economy [90]. There has also been increasing interest in the role of the circular economy [91] and of a potential role of universal basic income [92] or carbon pricing [93]. New research approaches are also attempting to bring together health, economics and environment to inform how we develop an economy that is conducive to wellbeing within planetary boundaries [94]. Further review evidence is required to inform these diverse approaches and practices.

### Implications for practice

The results of this study provide a simple yet comprehensive framework to support policy-makers and practitioners in addressing the macroeconomic determinants of health. Implementing these recommendations may require policymakers to pay attention to the power relationships which influence action on the commercial determinants of health [95], to collaborate across economic development and public health work [96], and to communicate the ways in which upstream factors impact on outcomes [97]. Based on the evidence reviewed here, the following recommendations can be made.

Governments can fund generous welfare states and social protection with careful attention to the evidence base for specific interventions. They should monitor health and inequality impacts of any economic transitions and aim to manage the pace of economic transitions to minimise the risk posed by sudden transitions. They should promote policies that reduce the risk of economic crises and mitigate the negative impact of these where possible. They should promote employment, good contractual conditions and occupational safety, and mitigate any potential negative impacts of unemployment or poor-quality work on health for example through welfare provision.

Governments should consider the risks of privatisation. Market regulation aimed at increasing consumer prices of health-harming products by at least 10–20% should be considered, particularly in conjunction with subsidies aimed at reducing price to consumers for health-promoting products.

Though conflicted, evidence suggesting a threshold beyond which GDP is not associated with positive health outcomes should be taken into account as it is possible that policies that pursue increases in national income may not result in major improvements in health or reductions in inequalities in developed countries. In the absence of clarity on this topic, countries should pay attention to the type of development that accompanies increases in national income, and to economic inequality at a macro level. This would also strengthen the case for measuring social progress in a broader way than simply GDP.

Whilst the review did not enable the identification of causal mechanisms, policymakers and practitioners can try to monitor the causal pathways through which economic factors affect health and health inequalities as well as the end outcomes.

### Future directions for macroeconomics and health research

This review identifies a cross-cutting need for more research in low- and middle-income countries. There is generally a need for more robust theoretical and conceptual work to underpin further research in this field including the causal mechanisms, and more work to understand how different populations are affected. Methods that take into account reverse causality, multilevel aspects of economic factors, of greater quality and over longer time periods are desperately needed. This is likely to take the form of natural experiments in many instances but could also include developing quantitative models that can estimate or forecast the impact of economic policies or factors on health outcomes. Much of the evidence base focuses on associations and more work is needed to inform interventions. This may include trial designs, and time series analyses of policy interventions.

This review supports the understanding of the macroeconomy as a complex system (see for example [98]) that leads to health outcomes, and the review provides a framework for how to consider this complex system within future health research. This has important implications for developing appropriate and targeted interventions and monitoring including intermediate and unintended outcomes. A complexity informed view of the economic determinants also supports an understanding of tipping points within the system – see for example [99]. Further work could include a more robust consideration of the intermediate steps along the causal chains and connections between economic determinants as a way to build empirically supported models of the macroeconomic determinants and the ways in which they influence health.

This review has identified several areas for research, including areas which have an existing evidence base and where questions remain, and areas which have no existing evidence base at the systematic review level.

Around market regulation, further empirical evidence is required for food and beverage taxation and for specific trade policies. Future systematic reviews should consider the role of competition, the consideration of externalities in pricing, production-level subsidies and fiscal measures for goods other than alcohol, food and tobacco.

The absence of retrieved evidence around the role of institutions in regulating the economy highlights the need for further systematic reviews in this area, including the role of central banks, banks, mortgages, startups, international organisations.

In terms of finance, there is a need for systematic reviews to consider the role of interest rates, inflation, deflation, monetary and fiscal policy, structural adjustment policies, international lending, foreign aid, financial transactions taxes and capital controls.

In terms of the labour force, further research is required to understand the impacts of paid maternal leave, gender inequalities around employment and welfare policies to mitigate unemployment. There is a need for systematic reviews around firm governance and ownership structures, the role of trade unions, minimum wage policies and labour force structure. Industry-specific research is also needed to inform policy and practice recommendations given the diversity of economic sectors. Future systematic reviews could consider the role of innovative interventions such as universal basic incomes, and universal basic services. Trends such as automation will likely present major challenges to research in this topic and lead to major societal changes which will also need to be studied.

There is a need for further research to understand the impacts of the social economy and welfare interventions at a population scale, and to expand the evidence on privatisation. Further systematic reviews could explore the impact of land tenure and property rights, informal economies, cooperatives, tax avoidance and government expenditure.

There is a need for more research to better understand the dynamics of GDP and health outcomes; and especially whether there is a threshold beyond which this relationship changes as this would have major implications for practice. Research needs to build on existing descriptive evidence around inequality and deprivation to move towards an intervention focus with clearer geographical units of analysis. Further systematic reviews can consider the role of industrialisation and aggregate productivity.

There is a need to improve the categorisation of exposure and effect in research around political economy type macroeconomic factors, and for systematic reviews of regional economic approaches.

Addressing these areas will require transdisciplinary programmes of research linking economists, public health researchers and sociologists (amongst others). It seems likely that large research funding will need to shift towards these upstream determinants. Likely priorities in terms of macroeconomic determinants would be to:

1. More fully characterise the macroeconomic determinants, especially
  i. What the health and inequalities impacts of underexplored macroeconomic factors are, including new economic practices. This will likely include significant theoretical and empirical work.
  ii. How the macroeconomic determinants impact on each other.
  iii. How they influence micro-economic determinants, intermediate variables and end outcomes. This will support further research and practice given the complex causal chains at play and the difficulty of measuring end outcomes given time lags.
2. Develop better evidence on interventions to target the macroeconomic determinants of health, particularly considering how these interventions can be implemented at scale.
3. Bring together new methodologies to support research and practice around the macroeconomic determinants, including automated ways of making sense of data and research, modelling techniques including multilevel models, supply chain analyses, systems mapping, political economic analyses, natural experiments, etc.
4. Bring together the fields of economics, health and sustainability to envision a healthy and sustainable economy and understand the required pathways to achieving it. One related such project may be to systematically explore the economic determinants of environmental sustainability, for example.

### Strengths and limitations

This is the first umbrella review to formally define and conceptualise the macroeconomic determinants of health, and to summarise systematic review-level evidence across all relevant fields and provide a summary of evidence gaps. The broad approach taken is a strength as it supports a holistic understanding of the economy as a complex system. The conceptual model developed for the review provides a coherent framework which can be used in future. The umbrella review methodology combined with AMSTAR has allowed the review to retrieve a range of evidence to build a clear picture of the macroeconomic determinants of health, building on existing systematic reviews. The review has also identified clear areas where evidence exists, and clear gaps in the systematic review level evidence. These are major contributions to the field.

However, there are some key limitations. Firstly, as with all umbrella reviews, summarising the key results from primary papers inevitably involves losing nuance and doing this at an umbrella review level involved an added risk over and above a standard systematic review especially as some of the included reviews are already reviews of reviews. This included, for example, original reviews not always including information on which of their prespecified outcomes were relevant to their findings which has made it difficult to consistently report specific outcomes in this umbrella review. To mitigate this risk a structured process was followed to synthesise the data from the original reviews into key themes for each category. Whilst data extraction was checked, the fact that double data extraction was not feasible provides another limitation of the systematic review.

Second, as this review has not drilled down to the level of individual primary studies, one concern could be that several reviews cover several of the same individual studies, thus leading to the potential for overemphasising the research findings through double counting. As far as possible, this issue was mitigated by synthesising the overarching key links made in the evidence base rather than emphasising the number of reviews and underlying studies – for example, it is possible when drawing from a range of reviews to decide whether the evidence is broadly positive, negative, mixed or inconclusive. It should also be noted that where reviews do cover similar studies, these reviews providing similar conclusions provide a validation of the reviews in their interpretation of the evidence base and this can be considered proof of reproducibility of the reviews though this does not change the strength of the underlying evidence. Where possible we have reported in Additional file 5 information regarding the quality of specific studies as described in reviews, and the methods used in underlying studies. However, there was significant heterogeneity in (i) the ways in which the included reviews assessed and discussed quality and (ii) the extent to which the methods of underlying studies were described in reviews. As such it has not been possible to provide a coherent summary of the quality of underlying evidence.

Third, although the robust component-based conceptualisation of the economy to design the search strategy was a key strength of this review, alternate views of the economy have adopted other framings reflecting the contested nature of economic theory and study (see for example a sector based view by Schafran et al [100]). It is not possible to discuss the specific health impacts of different economic sectors as the search was neither structured in this frame nor was it specifically designed around sectoral search terms. There are also several examples where debate around the conceptualisation used could shape the future of what is considered a macroeconomic determinant of health – for example our conceptualisation of market regulation did not include bans on smoking in public places as these shaped the pattern of end user consumption and only indirectly shaped purchasing, though this could be contested. The conceptualisation of the economy used as a basis for this review and the simplified model developed based on this review cannot be considered robust theories that explain the functioning of the economy; however they provide an important framework for examining the different subcomponents of the macroeconomy from a health perspective, and to reveal the current state of the evidence base around the macroeconomic determinants of health. It is hoped that this review can provide a framework for conceptual clarity and methodological coherence, and future research will likely involve critical discourse that refines and contests the scope and structure of the field.

Fourth, the explicit requirement for all included reviews to include both a population-level economic exposure and a health outcome means recommendations for practice can be provided from the evidence though it is not possible to provide robust evidence about intermediate causal steps within the pathway. Due to the focus on population-level exposures, the findings must be considered in light of other studies focusing on individuals; this is particularly true for employment and welfare where there is a substantial evidence base outside the scope of this review. For example, there is a recent umbrella review of welfare states which found only 6 studies [101]. The focus on population-level factors has also meant that included reviews included a considerable number of ecological and associational studies which limits the ability to definitely determine causality compared to intervention studies, especially in the context of the likely two-way relationship between health and the economy and the likelihood that impacts occur after a significant time lag. This presents a major challenge for future research into macro-level determinants of health. The complexity of exposures used in the retrieved reviews is another limitation – for example, definitions of economic crisis included decreases in unemployment and thus it has not always been possible to totally disentangle these different exposures.

Fifth, the use of a 10% second screen for the title and abstract phase and the high number of reviewers are limitations, as these increase the likelihood of human error or subjective interpretation of the inclusion criteria but were pragmatic given the need to screen over 15,000 results. Citation follow-up on key reviews will hopefully mitigate the risk that any major relevant reviews have been missed. Screening required expert judgement given the complex boundaries around the macroeconomic determinants. This added a degree of subjectivity, which is expected in a review of such a complex field. This is likely to be indicative of the challenges of using systematic review methodology for such a broad topic where the objective is not to answer a single tightly defined question. Relatively high percentage agreement across screeners both for the first phase of title and abstract screen with 10% check and for the second phase of dual full text screen are reassuring features. Similarly, the lack of dual data extraction is a weakness and although data extractions were checked when drafting the manuscript and in preparing the additional files, it is possible that elements of the original reviews have not been included in this review.

Sixth, the lack of consideration of grey literature retrieval or contact with authors and the lack of inclusion of non-English language papers may have affected the findings – especially in terms of low and middle income countries. It has not been feasible to conduct a thorough contextualisation of the findings of this review in the broader evidence base; such an attempt would be subjective and selective at best. Instead we have chosen to report the findings and provide a high level contextualisation based on several high-level reviews of the evidence. Whilst the majority of reviews retrieved are low quality this does not mean that the underpinning primary studies in the field are also of low quality.

It is possible that this current review which we have conducted may not score as “high quality” against AMSTAR2 criteria, despite our full awareness and best efforts to meet this where possible. Currently, it is more challenging to evaluate the quality of complex systematic reviews done on relatively higher order and/or upstream factors, such as economic determinants. The AMSTAR 2 does not include a systematic assessment of the quality of underlying evidence, so this was dependent on the original reviews which had varying approaches to quality and bias. This tool provided some differentiation to the included reviews though it may bias the evidence base towards evidence from health-related fields which are more likely to adhere to systematic review methodology. Issues that repeatedly led to a low AMSTAR 2 scoring included the non-registration of protocols and a lack of assessment of bias. That non-registration of protocols has played such a prominent part in reviews scoring lower on AMSTAR 2 may have led to an under-estimate of the quality of the evidence as this criterion does not necessarily reflect the validity of methods and analysis deployed by review authors.

## Conclusions

This review set out to provide a summary of the evidence base around the macroeconomic determinants of health or health inequalities and interventions to address these. This review has provided one grounded conceptualisation of these, and retrieved a broad range of evidence providing substance to this framework. It thus provides clear recommendations for policy and practice, and for research.

Employment and working conditions are important determinants of health and gender-based health inequalities. Evidence suggests that market regulation of tobacco, alcohol and food is likely to be effective at improving health and reducing inequalities in health including strong taxation, or restriction of advertising and availability. Identified risks to health outcomes include privatisation of utilities and alcohol sectors, income inequality and large area deprivation, and economic crises. Left of centre governments and welfare state generosity may have a positive health impact, but evidence on specific welfare interventions is mixed. Trade was found to have a mixed effect. There were no systematic reviews of the health impact of monetary policy or of large economic institutions such as central banks and regulatory organisations. Further research is needed into the macroeconomic determinants in general, especially in low and middle income countries.

This review sets out a comprehensive research agenda for people wishing to conduct further research on the macroeconomic determinants of health and interventions to improve health that target them. It also points to several effective intervention points for practice.

## Supplementary information

**Additional file 1.** PRISMA checklist.

**Additional file 2.** Search strategy for Medline.

**Additional file 3.** Screening tool.

**Additional file 4.** Data extraction form.

**Additional file 5.** Table of included papers.

**Additional file 6.** Table of papers excluded at full text stage and reasons.

## Data Availability

The original search results retrieved during the current study are available from the corresponding author on reasonable request. The list of papers that were full text screened and the list of papers included in the review are included in this published article [and its supplementary information files].

## Abbreviations

AMSTAR 2: Assessment of Multiple Systematic Reviews 2
DARE: Database of Abstracts of Reviews of Effects
GDP: Gross Domestic Product
JEL: Journal of Economic Literature

## Footnotes

1 The Preston Curve plots life expectancy against per capita GDP. In its original form, for data from 1900, 1930 and 1960, it demonstrated a positive relationship between wealth (in per capita GDP) and life expectancy (see [71]).
