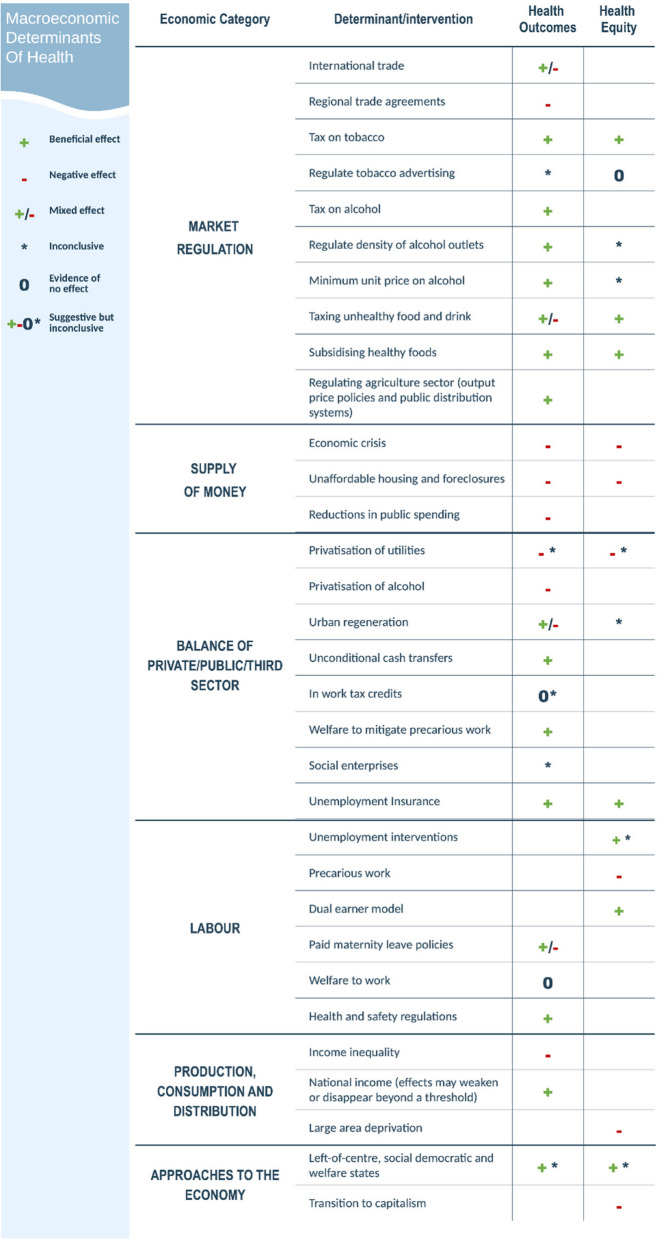# Correction: Going upstream – an umbrella review of the macroeconomic determinants of health and health inequalities

**DOI:** 10.1186/s12889-023-17273-4

**Published:** 2024-01-02

**Authors:** Yannish Naik, Peter Baker, Sharif A. Ismail, Taavi Tillmann, Kristin Bash, Darryl Quantz, Frances Hillier-Brown, Wikum Jayatunga, Gill Kelly, Michelle Black, Anya Gopfert, Peter Roderick, Ben Barr, Clare Bambra

**Affiliations:** 1 Leeds Teaching Hospitals NHS Trust, Beckett St, Leeds, LS9 7TF UK; 2 Department of Public Health and Policy, University of Liverpool, 3Rd Floor, Whelan Building, Brownlow Hill, Liverpool, L69 3GB UK; 3 Global Health and Development Group, School of Public Health, Imperial College London, St. Mary’s Campus, Norfolk Place, London, W2 1PG UK; 4 Department of Global Health and Development, London School of Hygiene and Tropical Medicine, 15-17 Tavistock Place, London, WC1H 9SH UK; 5 Department of Primary Care and Public Health, Imperial College London, Reynolds Building, St Dunstans Road, London, W6 8RP UK; 6 Institute for Global Health, Centre for Global Non-Communicable Diseases, University College London, 30 Guilford Street, London, WC1N 1EH UK; 7 School of Health and Related Research (ScHARR), The University of Sheffield, Regent Court, 30 Regent Street, Sheffield, S1 4DA UK; 8 NW School of Public Health, Health Education England North West, First Floor Regatta Place, Brunswick Business Park, Summers Road, Liverpool, L34BL UK; 9 Department of Sport and Exercise Sciences, Durham University, 42 Old Elvet, Durham, DH1 3HN UK; 10 Institute of Health Informatics, University College London, 222 Euston Road, London, NW1 2DA UK; 11 Junior Doctor and National Medical Director’s Fellow, London, UK; 12 Faculty of Medical Sciences, Newcastle University, Sir James Spence Building, Royal Victoria Infirmary, Newcastle Upon Tyne, NE1 4LP UK

**Correction: BMC Public Health 19, 1678 (2019)**

**https://doi.org/10.1186/s12889-019-7895-6**

The original publication of this article [1] contained an error in Fig. 3. The value "regulate tobacco advertising" in Fig. 3 should have been “0” (evidence of no net effect) for the impact on health equity, in line with the statement "restricting advertising was likely to have a neutral equity impact" of the results section. The incorrect and correct figure are shown in this correction article.

Incorrect Fig. 3


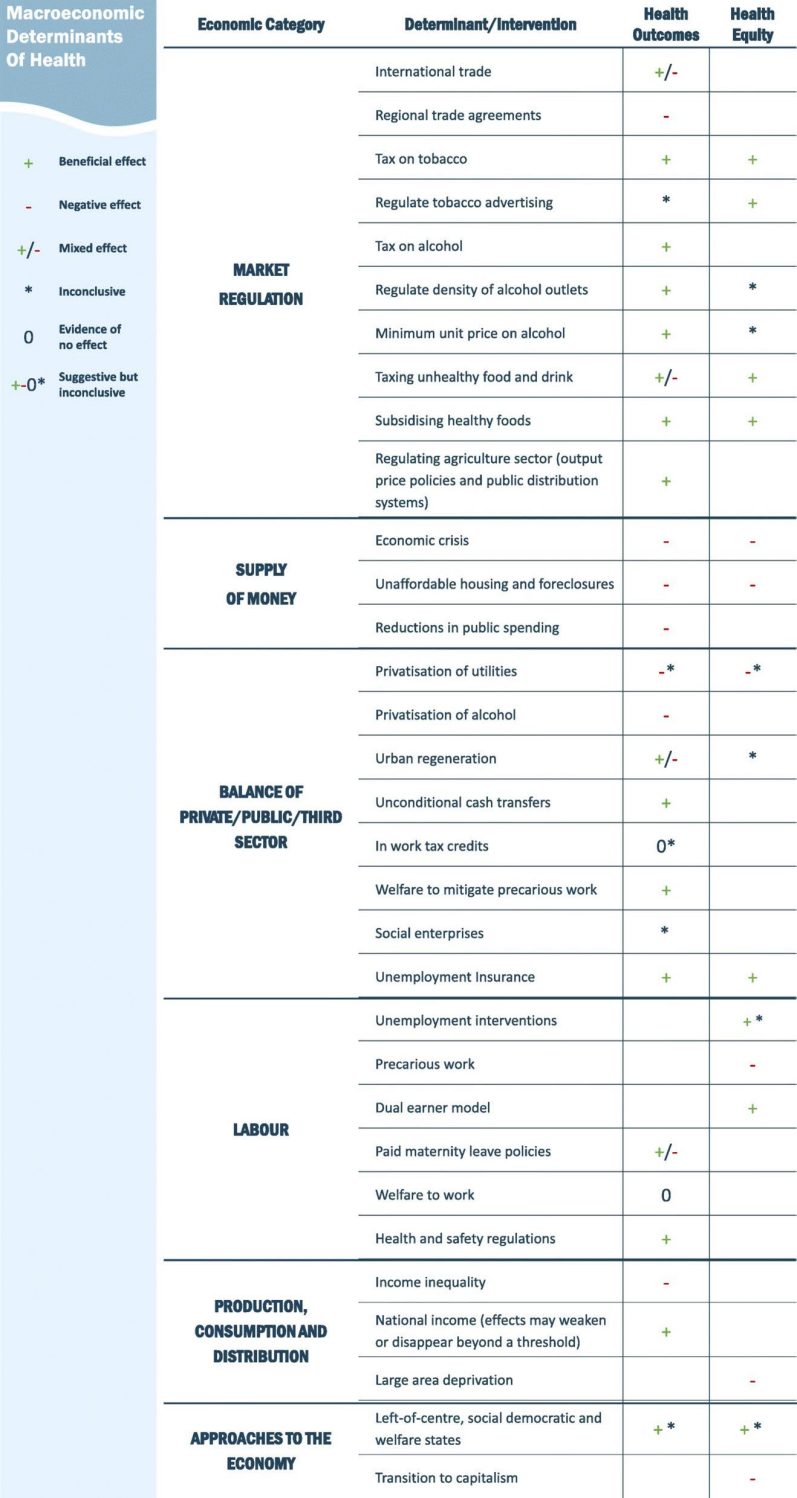


Correct Fig. 3