# Supplementary material for: Going upstream – an umbrella review of the macroeconomic determinants of health and health inequalities
Source: BMC Public Health. 2019 Dec 17;19:1678. doi: 10.1186/s12889-019-7895-6 (PMC6915896; doi:10.1186/s12889-019-7895-6)
Supplement: Supplementary file 2 — Additional file 2. Search strategy for Medline. [file 12889_2019_7895_MOESM2_ESM.docx]

|  | **Additional file 2 – Search strategy for Medline** |
| --- | --- |
| 1 | (health or wellbeing or well-being or mortality or "life expectancy" or morbidity or disease or incidence or prevalence or illness or death).mp. [mp=title, abstract, original title, name of substance word, subject heading word, keyword heading word, protocol supplementary concept word, rare disease supplementary concept word, unique identifier, synonyms] |
| 2 | Health Status/ |
| 3 | Health Status Disparities/ |
| 4 | Morbidity/ |
| 5 | Mortality/ |
| 6 | Incidence/ |
| 7 | Prevalence/ |
| 8 | or/1-7 |
| 9 | (Econom* factor or Econom* determinant or Econom* growth or econom* development or macroeconom* or macro econom* or economic integration or economic globalization or economic globalisation or Transitional Econom*).tw. |
| 10 | (market structure or market design or pricing or freemarket or free market or market* Competition or Monopoly or Oligopoly or financial markets or trade policy or international trade or international factor movements or international business or Remittances or international finance or financial transactions tax or taxation or tax evasion or evasion of tax or tax avoid* or marketing or advertising or antitrust or trade or business cycle or business fluctuation or remittances or externalities).tw. |
| 11 | (economic institutions or multinational firm* or central bank* or Banking or banks or depository institutions or business economics or IMF or WTO).tw. |
| 12 | (money supply or credit supply or supply of credit or interest rate* or rate* of interest or financial polic* or financialisation or financialization or financial services or financial institutions or financial cris* or corporate governance or corporate finance or fiscal or lending or debt or micro finance or mortgage* or monet* or inflation or deflation or structural adjustment or trade deficit or budget* deficit or investment or economic recession or currenc* or price level or monetary or international lending or foreign aid or national budget or national deficit or national debt or capital).tw. |
| 13 | (Scope of Government or Social Security or underground economy or welfare programs or entrepreneurship or non profit or nonprofit or informal econom* or land ownership or land reform or shadow econom* or informal econom* or alternative econom or informal sector or urban econom* or regional econom* or rural econom* or Nationalization or Nationalisation or Privatisation or Privatization or Government Expenditure* or Size of Government or social enterprise* or public enterprise* or private enterprise* or Land Ownership or ownership of land or Land Tenure or Land Reform or public investment or Property rights or Open Econom* or subsid* or public good or cooperative enterprises or Welfare state).tw. |
| 14 | (Firm Objectives or objectives of the firm or objectives of firms or organization of firms or organization of the firm or organisation of firms or organisation of the firm or Firm Organization or Firm Organisation or Firm Behavior or Firm Behaviour or behaviour of firms or behaviour of the firm or behavior of firms or behavior of the firm or retirement or compensation package* or trade union or labor managed firm* or labour managed firm* or Worker* Rights or rights of workers or employee managed firm or employee owned Firms or firm performance or wage* or Human Capital or income* or employment or unemployment or enterprises or entrepreneur* or labor demand or labour demand or demand for labor or demand for labour or labor economics or labour economics or labor supply or labour supply or supply of labor or supply of labour or labor discrimination or labour discrimination or cost of labor or cost of labour or labor cost* or labour cost* or labor mobility or labour mobility or labor market or labour market or labor standards or labour standards or labor force size or labour force size or size of the labor force or size of the labour force or labour force structure or labour force structure or structure of the labor force or structure of the labour force or labor management relations or labour management relations).tw. |
| 15 | (resource distribution or distribution of resources or economic justice or externalit* or Gross Domestic Product or gross national income or industrialisation or industrialization or industrial structure or industrial policy or industrial ecology or poverty or wealth or economic inequalit* or production of goods or production of services or means of production or consumption of goods or consumption of services or pattern* of consumption or productivity or manufacturing or startups or social status).tw. |
| 16 | (socialist or socialism or Public Economics or Welfare Economics or environmental economics or ecological economics or Marx* or Keynes* or Neoclassic* or capitalism or capitalist or neoliber* or political economy or economic austerity or (economic recession or degrowth)).tw. |
| 17 | Socioeconomic Factors/ |
| 18 | Income/ |
| 19 | Employment/ |
| 20 | Poverty/ |
| 21 | Social Class/ |
| 22 | Economics/ |
| 23 | or/9-22 |
| 24 | and/8,23 |
| 25 | Meta-Analysis as Topic/ |
| 26 | meta analy$.tw. |
| 27 | metaanaly$.tw. |
| 28 | Meta-Analysis/ |
| 29 | (systematic adj (review$1 or overview$1)).tw. |
| 30 | exp Review Literature as Topic/ |
| 31 | or/25-30 |
| 32 | cochrane.ab. |
| 33 | (medline or embase).ab. |
| 34 | (psychlit or psyclit).ab. |
| 35 | (psychinfo or psycinfo).ab. |
| 36 | (cinahl or cinhal).ab. |
| 37 | science citation index.ab. |
| 38 | bids.ab. |
| 39 | cancerlit.ab. |
| 40 | or/32-39 |
| 41 | reference list$.ab. |
| 42 | bibliograph$.ab. |
| 43 | hand-search$.ab. |
| 44 | relevant journals.ab. |
| 45 | manual search$.ab. |
| 46 | or/41-45 |
| 47 | selection criteria.ab. |
| 48 | data extraction.ab. |
| 49 | 47 or 48 |
| 50 | Review/ |
| 51 | 49 and 50 |
| 52 | Comment/ |
| 53 | Letter/ |
| 54 | Editorial/ |
| 55 | animal/ |
| 56 | human/ |
| 57 | 55 not (55 and 56) {No Related Terms} |
| 58 | or/52-54,57 |
| 59 | 31 or 40 or 46 or 51 |
| 60 | 59 not 58 |
| 61 | 60 and 24 |
