# Supplementary material for: Going upstream – an umbrella review of the macroeconomic determinants of health and health inequalities
Source: BMC Public Health. 2019 Dec 17;19:1678. doi: 10.1186/s12889-019-7895-6 (PMC6915896; doi:10.1186/s12889-019-7895-6)
Supplement: Supplementary file 3 — Additional file 3. Screening tool. [file 12889_2019_7895_MOESM3_ESM.docx]

**Systematic review screening tool**

IF YES GO TO Q2, IF NO EXCLUDE‬

IF YES GO TO Q5, IF NO EXCLUDE‬
