# Supplementary material for: Going upstream – an umbrella review of the macroeconomic determinants of health and health inequalities
Source: BMC Public Health. 2019 Dec 17;19:1678. doi: 10.1186/s12889-019-7895-6 (PMC6915896; doi:10.1186/s12889-019-7895-6)
Supplement: Supplementary file 4 — Additional file 4. Data extraction form. [file 12889_2019_7895_MOESM4_ESM.docx]

| **Unique ID** |  |
| --- | --- |
| **Title** |  |
| **Authors** |  |
| **Journal/Source** |  |
| **Category of determinant** |  |
| **Extracted by (initials)** |  |
| **Is this a key review? (complete once you have an overview of the category)** |  |
| **Review characteristics** | **Review characteristics** |
| • Key population-level economic determinants identified in review and their conceptual synonyms | Primary |
|  | Secondary |
| • Is this an economic characteristic, strategy, policy or intervention? | At the individual level |
|  | At the population level (NB if blank, discuss with lead author as may need to exclude) |
| - How is the exposure to the economic determinant measured? |  |
| • Outcomes used (specified by review rather than results found) | At the individual level |
|  | At the population level |
| • Population |  |
| • Setting (Low, Middle or High Income countries) |  |
| • Number of studies included in review |  |
| • Number of professional/peer reviewed databases searched and disciplines |  |
| • Was grey literature search, contact with authors or citation follow-up carried out? (which one?) Include grey literature sources. NB if blank, discuss with lead author as may need to exclude |  |
| • Types of studies included |  |
| • Synthesis methods—narrative/meta-analysis/realist/etc. |  |
| • Time/language restriction |  |
| **Results of review** | **Results of review** |
| • Main results and strength of findings (size of effect) including variations by gender, ethnicity or socio-economic status  Extract only relevant findings to scope of our umbrella review (ie health outcomes, economic determinants etc)  Focus on review level findings not individual studies |  |
| • Quality of underlying evidence: risk of bias and confounding, consistency across multiple settings. |  |
| • Proposed mechanistic pathways ie how does the review propose that the economic determinant influences heatlh outcomes |  |
| • Clear evidence gaps identified |  |
| • Key contextual factors (if the review identifies political/social/historical or other factors which have influenced the effect of the economic determinant, or which provide the background in which the economic determinant acts) |  |
| **Citations extracted (if key review only)** |  |
| **Modified AMSTAR 2**  **Complete separate AMSTAR 2 checklist, any N/A in AMSTAR is fine. Look at supplementary data for review**  **Count weaknesses**  Critical weaknesses: Items 2, 4, 9, 11 if applicable, 13, 15 – partial yes still counts as critical weakness.  Non critical weaknesses are all others  **Guideline for grading see box 2. Below**  For any queries with AMSTAR 2 questions see original AMSTAR 2 paper  <http://dx.doi.org/10.1136/bmj.j4008> | Critical weaknesses? |
|  | Non-critical weaknesses? |
|  | AMSTAR grading: High/medium/low/critically low confidence? |

| Box 2: Rating overall confidence in the results of the review |
| --- |
| • High   - No or one non-critical weakness: the systematic review provides an accurate and comprehensive summary of the results of the available studies that address the question of interest - •   Moderate - •   More than one non-critical weakness*: the systematic review has more than one weakness but no critical flaws. It may provide an accurate summary of the results of  the available studies that were included in the review - •   Low - •   One critical flaw with or without non-critical weaknesses: the review has a critical flaw and may not provide an accurate and comprehensive summary of the available studies that address the question of interest - •   Critically low - •   More than one critical flaw with or without non-critical weaknesses: the review has  more than one critical flaw and should not be relied on to provide an accurate and  comprehensive summary of the available studies  *Multiple non-critical weaknesses may diminish confidence in the review and it may be appropriate to move the overall appraisal down from moderate to low confidence. |
